# A review of measles control in Kenya, with focus on recent innovations

**DOI:** 10.11604/pamj.supp.2017.27.3.12118

**Published:** 2017-06-21

**Authors:** Kasidet Manakongtreecheep, Robert Davis

**Affiliations:** 1 Yale University, New Haven, Ct 06520, USA; 2 American Red Cross, Washington D.C, USA

**Keywords:** Measles, elimination, innovation, innovations, vaccination, epidemiology, immunization

## Abstract

Despite the existence of a highly effective measles vaccine and the decrease in worldwide deaths from measles by more than 79% from the 2000 baseline levels, measles today remains one of the leading causes of vaccine-preventable deaths in the world. The African region is a key player in the global fight against measles. Africa has made tremendous progress in its effort to immunize children and to control the disease, increasing its regional measles vaccination coverage from 56% in 2001 to 85% in 2010. The Republic of Kenya has been a strong follower of the World Health Assembly and Measles Elimination 2020 resolutions, which aims to eliminate measles from the country. Since the beginning of the 21st century, Kenya has faced many challenges, but also aid, in the form of new innovations, in their fight against measles. In 2002, Kenya started its first SIA using A-D syringes, and from 2003-2005, GAVI funded injection safety support (INS) to Kenya, as an effort to scale-up safe injection in sub-Saharan Africa. In 2016, the Kenya introduced Measles-Rubella (MR) combined vaccine in its nationwide SIA campaign, after recognizing that rubella is a disease that must be controlled along with measles. In 2009 and 2012 SIAs, Red Cross volunteers conducted H2H visits to promote immunization as well as document information from the community with regards to immunization, including the current coverage, to campaign management levels. Case-based surveillance, using real-time PCR, measles-specific IgM detection and Epi-link were used to confirm and map measles infection during outbreaks. Alternative serosurveys such as Dried Blood Spot and Urine sample surveys were also tested in Kenya. In 2013 and 2016, two studies were also conducted in Kenya on the use of SMS reminder system for routine immunization. These studies, which showed SMS to significantly improve the vaccination coverage, paved way for use of SMS in a larger scale in Kenya.

## Introduction

### Global

Measles is a deadly and highly infectious virus that has affected mankind for centuries. Despite the existence of a highly effective measles vaccine since 1963, and although vaccination has cut the number of worldwide deaths from measles by more than 79% from the 2000 baseline levels, measles today remains one of the leading causes of vaccine-preventable deaths in the world [1]. In 2001, the Measles & Rubella Initiative (MRI, formerly called the Measles Partnership) was formed by American Red Cross, the United States’ Centers for Disease Control and Prevention (CDC), the United Nations Foundation, UNICEF, and W.H.O., with the goal of reducing measles mortality by 90% by 2010, compared to 2000 baselines [2]. In 2008, the Global Immunization Vision and Strategy of the W.H.O. created a strategic framework for vaccination with the MRI goals in mind, which were achieved by most countries [3]. At the 2010 World Health Assembly (WHA), member states endorsed the plan for accelerated control of measles through national vaccination campaigns, in order to reduce mortality among children, in line with the Millennium Development Goal 4 [4]. The WHA global plan includes sets of targets to be achieved by 2015: ≥ 90% coverage with the first dose of measles containing vaccine nationally and ≥ 80% vaccination coverage in every district; reduction in annual measles incidence to <5 cases per million and maintenance of that level; and measles mortality reduction by 95% compared to the 2000 estimates [5].

At the 2012 World Health Assembly, the Global Vaccine Action Plan (GVAP) was adopted, with the mission of massively reducing vaccine-preventable deaths, which includes eliminating measles from 5 of the 6 W.H.O. regions, and increasing vaccination coverage even further to 95% by 2020 [6]. In 2015, the global measles coverage was at around 85% and the decline in mortality from measles at around 79%, compared to the 2000 baseline estimates. Both the figures were short of the WHA targets and at risk of missing the 2020 GVAP objectives. With all but one region likely to miss the GVAP target for regional measles elimination, measles is considered one of the areas furthest behind in the GVAP objectives [7]. In 2016, the Strategic Advisory Group of Experts (SAGE) gave a recommendation for the introduction of routine second dose vaccine in all countries, regardless of their fulfillment of the 80% coverage criterion for introduction of the two-dose schedule [8].

Even though the region of the Americas has eliminated indigenous measles transmission, cases of measles outbreak still occur in the U.S.A. and other countries, largely because of parental objections [9]. The anti-vaccination movements in the West and non-attainment of vaccination targets by many developing countries mean that measles is still very much a threat to humankind.

### Regional

The African region is a key player in the global fight against measles. With the most cases of measles at the turn of the 21st century, Africa has made tremendous progress in its effort to immunize children and to control the disease, increasing its regional measles vaccination coverage from 56% in 2001 to 85% in 2010 [10]. In 2008, Africa achieved the goal set by MRI, by reducing measles mortality by 92% 2 years before the deadline. After the WHA measles control resolution was announced in 2010, the WHO Regional Committee for Africa in 2011 announced a resolution, supported by all 46 Member States, for measles elimination by 2020 (ME 2020) [11].

In recent years however, the Africa region has experienced measles outbreaks and stagnation in vaccination coverage. The outbreaks came as a result of conflicts in the region disrupting the supplemental immunization activity (SIA) efforts, of resistance to vaccination from religious groups, and from the epidemiological shift in measles cases towards older age groups [12]. Africa did not achieve the WHA target in 2015, and is at risk of missing the ME2020 target [13]. Global partners must increase their effort and work with the national-level governments, to strengthen health systems and to implement high quality SIAs, in order to get back on track for measles elimination.

### National

The Republic of Kenya has been a strong follower of the WHA and ME 2020 resolutions, and has had continuous SIAs since 2002. However, the delay in the 2006 SIA resulted in a massive outbreak of measles, which was a setback in achievement of national and international goals [14]. Due to Kenya’s border with the conflict countries of Somalia and South Sudan, Kenya faces a large influx of refugees and immigrants, many not vaccinated against measles. This has led to several outbreaks in the past few years, either in refugee camps or in informal communities [15]. In 2013, Kenya introduced a second dose measles vaccine in its routine immunization schedule, but has not had high MCV1 or MCV2 coverage in recent years [16]. In May 2016, the Kenyan government launched an <15 measles and rubella campaign to increase its coverage and introduce a combined measles-rubella (MR) vaccine to the country. It was also announced that MR vaccine will be formally introduced into the routine immunization schedule in early 2017 [17]. It remains to be seen whether the addition of second dose vaccine and MR vaccine into the routine schedule will lead to an improvement in measles coverage and incidence decline in the country.

## Methods

This article outlines the situation of measles in Kenya from the pre-vaccination era to 2016, and highlights any significant events, innovations or policy changes that has occurred since 2000. Information on this article are found from online peer-reviewed publications, from reports and presentations from international non-governmental organizations, from government reports and policies, and from personal communications with researchers on their unpublished research. Innovations are categorized into (1) Past innovations, or innovations that has been implemented in the past in Kenya, and is either still in-use or has been replaced by newer protocols or innovations; (2) Current innovations, or innovations that are already in the process of launching or implementing throughout the country, either as pilot projects or full-scale country-wide launch and; (3) Future innovations, or innovations that are still under development but hold high potential for its future use in Kenya. Although this article is by no means an exhaustive source on measles in 21st century Kenya, the article aims to be as comprehensive a resource as possible for readers of all levels. This version of the article focuses on the innovations. For a full-length article, please visit: http://childsurvival.net/.

### Past innovations in measles control in Kenya

### Vaccine quality and injection safety

Measles vaccination programs would benefit from a vaccine that can improve delivery methods, decrease cost and manpower, simplify logistics, and increase safety. Current subcutaneous injection vaccine requires specially trained health workers to reconstitute the vaccine, administer the vaccine, and dispose of the needles and syringes safely. Furthermore, the storage of the vaccine and its diluent at cold temperatures using cold chain requires space and facility that could accommodate it, and reconstituted vials must also be used within 6 hours or discarded [18]. This leads to vaccine wastage and increases the cost. The lack of skilled health workers and the difficulty in storage of vaccines, as well as the increase risk of disease transmission through needle re-use, has been a limiting factor in many developing countries’ fights against measles [19].

### Autodisabled syringes and bundling

**Figure 1:**
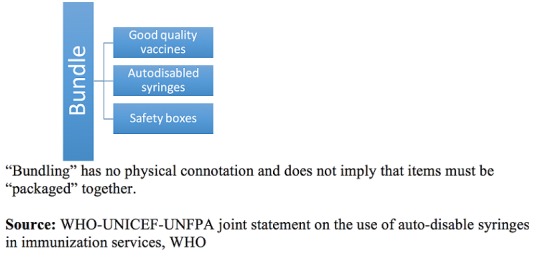
The term “bundling” has been chosen to define the concept of a theoretical “bundle”

In 2000, reuse of disposable syringes has caused more than 22 million infections and making 39% of all injections unsafe [20]. In 2001, to avoid reuse of syringes, all measles SIAs funded by MRI were conducted using safe injection materials, which include UNICEF recommended “bundling” of vaccination materials: autodisabled (A-D) syringes, reconstitution syringes, and safety boxes (Figure 1) [21]. In 2002, Kenya started its first SIA using A-D syringes, and from 2003-2005, GAVI funded injection safety support (INS) to Kenya, as an effort to scale-up safe injection in sub-Saharan Africa. In 2004, AD syringes became available in all districts of Kenya, and continue to be used for all SIAs and routine immunization even after the MRI funded SIAs and GAVI INS funding ended [22] (Figure 1): “Bundling” has no physical connotation and does not imply that items must be “packaged” together. Source: WHO-UNICEF-UNFPA joint statement on the use of auto-disable syringes in immunization services, WHO.

### Aerosolized vaccines

Aerosolized vaccines for measles have been suggested and developed since the 1980s, as a needle-free alternative to subcutaneous injection vaccines [23]. Although current developing aerosolized vaccines against measles have been immunogenic, they were however, found to be inferior to the subcutaneous vaccine in terms of seropositivity for younger age group. For children below 10 months, the seroconversion rate was lower with aerosol than with subcutaneous delivery, and for children between 10-35 months, the pooled seroconversion rate was 93.5% with aerosolized vaccine and 97.1% with subcutaneous vaccine. Only in children 5-15 years of age were there better seroconversion rates in aerosolized vaccines [24]. Most recently, aerosolized vaccine development suffered another setback when a randomized controlled trial of more than 2000 children was run in 2015, showing that 85.4% of children receiving the aerosol vaccine had sufficient antibody levels, compared to 94.6% with sufficient antibody levels for protection through subcutaneous injection [25]. This means that aerosol vaccination still could not achieve the required 95% protection that is required for herd immunity [26].

### Second dose measles vaccine introduction

**Figure 2:**
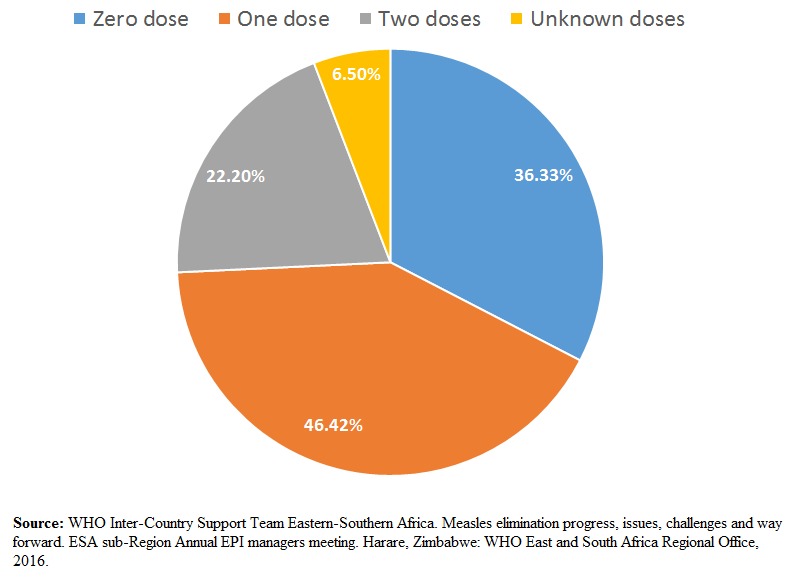
Vaccination status of confirmed measles cases; 2015; Kenya

Because the median proportion of measles vaccinated infants aged 8-9 months who seroconverted is only at 89.6%, and primary vaccination failure actually occurs in up to 15% of infants vaccinated at age 9 months, a second catch-up dose was proposed [27]. Studies on revaccination in children who did not seroconvert after their first dose of measles vaccine (MCV1) showed a median of 97% immunity after their second dose (MCV2) was administered [28]. A study of confirmed cases of measles in Kenya showed that the majority of the confirmed cases have actually been vaccinated with one dose, and that two doses of vaccination made up a much smaller portion of the measles cases (Figure 2).

**Figure 3:**
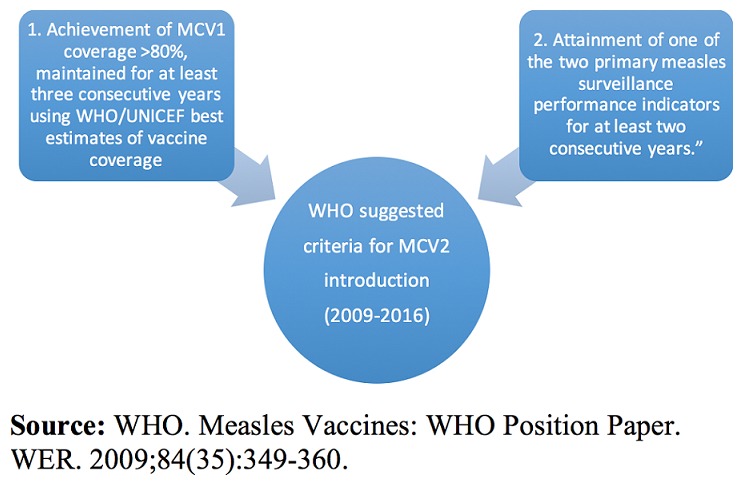
WHO suggested criteria for MCV2 introduction (2009-2016)

Through maintaining a satisfactory MCV1 coverage and attaining one of two primary measles surveillance performance indicators, Kenya qualified under the W.H.O. criteria for MCV2 introduction in 2013 [29]. The coverage criterion however, has been lifted in 2016 by SAGE Working Group on Measles & Rubella [30] (Figure 3).

Kenya is still early on in its MCV2 introduction. Therefore, SIAs must still be continued in order to control the number of susceptible children so as not to reach the critical community size and potentially trigger an outbreak [31]. The purpose of MCV2 in routine schedule is to decrease Kenya’s reliance on SIAs, which will reduce the frequency of SIAs needed and eventually cause all SIAs to stop once > 93% of the population immunity can be maintained with routine MCV1+2 schedule alone. It is also crucial to use MCV2 as a reminder for child health visits in the second year of life, linking it to other health interventions [32]. As for the optimal timing of routine delivery of MCV2 in Kenya, it is suggested by the Ministry of Health to be at 18 months or first contact after 18 months, thus ensuring that there is at least a one-month interval between MCV1 and MCV2 vaccination [33]. When a child comes in very late for the first dose of vaccine, two doses of measles vaccination in total must still be given to the child, with MCV1 and MCV2 administered 1 month apart from each other [34].

Initially, MCV2 coverage in Kenya was low, with only 28% nationwide coverage in 2015 [35]. This is, however, consistent with the coverage of the African region, with low coverage experienced in other countries as well [36]. A study on the uptake of second dose of measles-containing vaccine among children in Kakamega county, Kenya, showed that the risk factors for the children missing MCV2 are the caretakers’ awareness of MCV2 and other routine vaccines, and the distance from a vaccination facility [37].

### Measles-Rubella vaccine introduction

Until May 2016, the Republic of Kenya used the standalone measles vaccine. On 16 May, 2016, the Kenyan government introduced Measles-Rubella (MR) combined vaccine in its nationwide SIA campaign. This change came as a result of an increase in cases of rubella in Kenya in the past few years, with 422 cases in 2015. Rubella can also have serious consequences for pregnant women, which include miscarriages, fetal deaths, still births and congenital rubella syndrome – a severe birth defect. On 11 November 2016, the Kenyan government introduced MR vaccine into the routine immunization schedule, replacing all current measles vaccine in stock with MR vaccine for all 47 counties.

## Current status of knowledge

### Current innovations in measles control in Kenya

### House-to-house social mobilization

The purpose of House-to-House (H2H) social mobilization as part of immunization activity is to educate residents about the importance of the vaccination campaigns. It also creates an important communication link for the community on routine immunization for sustainable measles and rubella control and elimination. The mobilization includes advocacy visits to community leaders, house to house sensitization (visits), public announcements (at events, markets, and places of worship), and media announcements (print and electronic) [38].

In Africa, H2H social mobilization has been used on a subnational basis in Red Cross supported campaigns in 18 countries, including Kenya, and in UN supported campaigns in 2 countries [39]. In 2013, WHO/AFRO and the Measles and Rubella Technical Advisory Group stated the following: “WHO AFRO is requested to. . . provide guidance for all countries to standardize their approach including, but not limited to. . .:“the implementation of house to house mobilization before and during SIAs in priority areas.” [40].

In Kenya, H2H sensitization is usually done by Kenya Red Cross volunteers, who communicate to caregivers the messages from the Ministry of Health. At each household, the Red Cross volunteer delivers the information regarding vaccination and makes a count of targeted children in the household in order to obtain demographic estimates. The volunteers are trained and can answer questions about measles and rubella vaccination. The volunteers themselves cannot administer the vaccine, and measles vaccine can only be received at health facilities. H2H visits are done by 2 volunteers, who are resident and known in the community [41]. In the 2009 Kenya measles SIA, districts with H2H sensitization showed higher administrative vaccination coverage than in districts without H2H, with 73% unweighted coverage in H2H districts compared to 61% in non-H2H districts [42]. The Lions Clubs of Kenya were also active in social mobilization, and were involved in creating immunization events around big cities’ health centres, which helped increase trust and demand for the vaccine, notably through the use of panel truck and advocacy toolkits [43].

In the 2012 SIA, Red Cross volunteers conducted pre-campaign H2H visits to promote immunization as well as document information from the community with regards to immunization, including the current coverage, to campaign management levels. The documentation was done in real-time by using a web-enabled mobile phone application (episurveyor) and the documented data were analysed daily in order to adjust service delivery plans in an evidence-based manner. Post-campaign house visits were also conducted to verify immunization and detect adverse events following immunization. A follow-up study was conducted to evaluate the effect of the house visits. Research showed that 25% of households would likely miss the vaccination if not for the house visit. The visit also helped reduce misconceptions, and opposition to vaccination due to fear of injections and trust in herbal remedies. The H2H visits were also listed as the most easily remembered sources out of all the promotional media on immunization [44].

### Case-based surveillance

Starting in 2002, Kenya implemented a system of case-based surveillance for measles, which included a case report form and blood test for measles IgM for each suspected measles patient who visited a health facility [45]. If five or more cases were reported per 100,000 persons from the same health area in a month, an outbreak is suspected. If three specimens from five cases tested positive, the outbreak is confirmed [46]. Untested cases are confirmed by epidemiologic linkage, and throat swabs are collected for viral genotyping [47].

In 2011, following an outbreak in Eastleigh, an ethnic Somali community in Nairobi, the Ministry of Public Health and Sanitation stepped up its active surveillance and analysis of surveillance information in order to identify all districts reaching the threshold for outbreaks. W.H.O. surveillance data showed a pattern of spread from Eastleigh to all of Nairobi and from Nairobi to most rural districts. This pattern of viral seeding is not unique to Kenya [48]. Red Cross enhanced its surveillance through sensitizing all health workers to be on high alert while conducting continuous intensive measles case search and to report all suspect measles cases as per the measles case definition, investigation, and reporting guidelines. Furthermore, the line list of all cases since the onset of the outbreak was updated and maintained on a daily basis with daily updated cases and deaths submitted to the Division of Disease Surveillance and Response (DDSR) daily. All suspected measles cases were referred for clinical and laboratory diagnosis, and serological analyses were done constantly with immediate feedback of lab confirmed cases for action [47-49].

**Figure 4:**
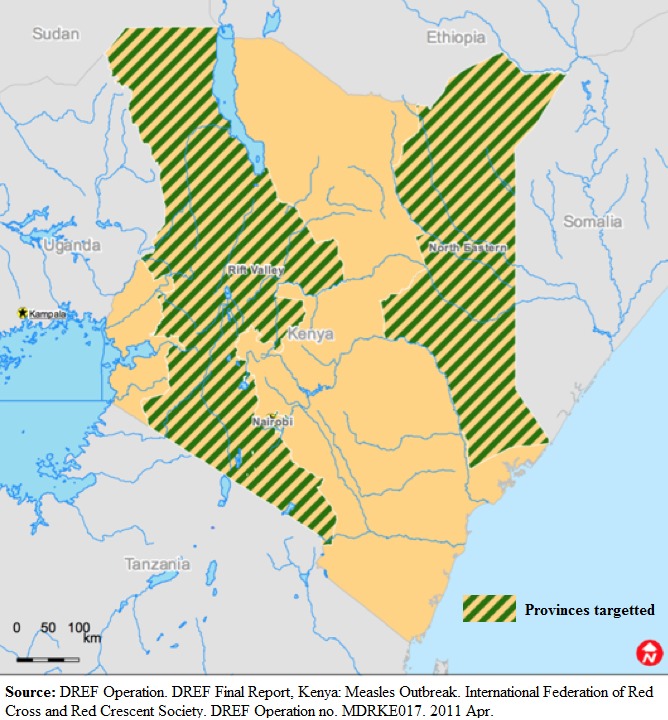
Map showing provinces targeted for case-based surveillance during the 2011 outbreak

Laboratory diagnosis is essential for confirming measles cases, especially when an outbreak is suspected. Real-time PCR and measles-specific IgM detection are the two most common methods for confirming measles infection. In Kenya, all suspected measles cases were referred for clinical and laboratory diagnosis at the Kenya Medical and Research Institute. In 2015, surveillance data were conducted using laboratory diagnosis and epidemiological linkage (Epi link) to find the susceptibility profile for measles in Kenya by age group (Figure 4).

### Seroepidemiological surveys and other assays

Seroepidemiological surveys (serosurveys) for measles surveillance have mostly been used in developed countries, although developing countries have used serosurveys to identify certain risk groups with low prevalence of immunity [50]. The challenges for developing countries are the limited access to high-quality laboratories and appropriate assays, and logistical challenges in conducting surveys that are representative of the populations, especially if venous blood samples, considered the gold standard, are required [51]. In Kenya, assays using a more easily accessible specimen of oral fluid through throat swabs showed that measles campaigns reduced susceptibility by 70% [52]. Dried blood spot (DBS) is a more accessible serosurvey specimen, since dried blood spot on filter paper is easier than liquid blood samples to store and transport to the laboratory, as well as being faster and safer to collect. Urine specimen has also been used as an alternative for serum in identifying genotype of the virus [53]. DBS, oral fluid and urine specimens can all be used for virus isolation or direct RT-PCR to detect RNA from measles virus, without needing specialized laboratory techniques and biosafety requirements that are not available throughout the laboratory network [54]. In measles surveillance, virus isolation and RNA detection are more likely to be successful if sample is obtained for lab confirmation within 3 days after onset of rash [55]. Serosurveys and other assays that measures antibodies, however, will detect both IgM and IgG a few days after rash onset, since antibodies are produced during the primary immune response. Sensitive ELISA assays have shown that IgM antibody levels peak after about 7-10 days and then decline rapidly, and IgG antibody levels peak after about 3 weeks and persist long after infection [56]. In a 2009 study in Kenya, DBS was used to obtain rubella seroprevalence rates in specific age and socioeconomic groups. A regression analysis model showed that older age groups have greater odds of having contacted rubella [57].

### SMS reminders

Short message service (SMS) reminders and incentives have been shown to improve health care-seeking behaviours, and have been recommended for application in routine and supplemental measles immunization activities. SMS can be used as a convenient and easily scalable way to inform caregivers of the disease and the importance of immunization, and then to remind them of vaccination schedules and campaigns in case of forgetfulness. SMS reminders have the potential to help increase vaccination coverage, as well as to increase the public’s positive image of vaccination activity [58].

In 2013, the feasibility of using either SMS reminders or conditional cash transfers (CCT) was tested in rural western Kenya. The study found that 90% of mothers in the SMS program received the SMS reminders, with 83% receiving their CCT through m-PESA, a paperless remuneration technology [59]. In 2014, a study was conducted in three counties of Kenya to evaluate the impact of SMS and sticker reminders to reduce dropouts from the measles vaccination program. SMS reminders were shown to lead to the lowest dropout rate of only 4%, compared to 16% among those who received sticker reminders and 17% among the control group that did not receive any reminders. This shows that SMS reminders can reduce vaccination dropout rates in Kenya [60]. In 2016, a study using cluster randomized control trial was done in Western Kenya, in order to assess the impact of mobile phone reminders and CCT as travel subsidies on improving childhood immunization coverage rates and timeliness. Research found that 86% of SMS-only participants, 86% of SMS+CCT (value of 75KES) participants, and 90% of SMS+CCT (value of 200KES) participants had their child fully immunized after the 12-month trial was over. This is a much higher immunization rate than for those in the control group (82%), showing that SMS reminders, with or without incentives, can become an alternative way to raise coverage in resource-constrained settings [61].

### SMS alerts during campaigns

In the 2016 SIA, research, led by the Ministry of Health and in collaboration with Kenya Red Cross and US Centers for Disease Control, was done in Kenya’s Western region to explore the feasibility of using the mobile phones to improve immunization nationwide. The study “sent multiple bulks of SMS reminder messages about the MR campaign (including messages informing the dates, locations, target group for vaccination, and importance of vaccine) through the Safaricom and Airtel networks in selected counties”. The analysis of the SMS alerts continues as of this writing (December 2016) and is likely to appear from Kenya in the new year.

### Possible future innovations

### Microneedle patch

Microneedles are micron-scale needles coated with a dry formulation of vaccine which dissolves in the skin and does not require reconstitution of vaccine or use of hypodermic needles. The microneedle patch (MNP) can be applied directly to the skin and left for 10 minutes, after which the patch can be taken off and discarded with very small risk for disease transmission from sharp objects due to its microscopic needles [62]. MNP has been shown to be immunogenic in non-human primates and can be stored for at least 30 days at room temperature without significant loss of viral titer [63]. These characteristics are important for creating a more effective and efficient vaccination method that could help increase coverage during immunization campaigns, especially in countries with many personnel, storage and financial limitations [64]. Future development of this new vaccine delivery system could see it being used instead of the traditional hypodermic needles in developing countries such as Kenya.

## Conclusion

In an evaluation of various innovations in communication technologies used in 11 urban districts during the 2012 Kenya SIA, positive results were shown for all the innovations, suggesting that scaling up some of these innovations will be very beneficial to the campaign. The adaptive planning and management of supplemental measles immunization activities based on real time evidence could be a key factor in increasing SIA coverage in the future. Improvement in technology will still be needed, especially in verifying children’s vaccination status in order to send the correct follow-up SMS. Better systems for a more accurate way of inputting date of birth in the data are also needed, in order to reduce human error. Unequal access to mobile phones among different income populations, lack of proper framing of SMS reminders, and language barriers also pose a challenge to the technology [44].

### What is known about this topic

- The Republic of Kenya has been a strong follower of the WHA and ME 2020 resolutions, and has had continuous SIAs for measles since 2002;
- The delay in the 2006 SIA, as well as Kenya’s migration problems, both with neighboring countries and between cities, resulted in a few outbreak of measles in the past 10-15 years;
- Kenya introduced a few campaigns and policy changes to try and improve measles vaccine coverage, including introducing second dose measles vaccine in its routine immunization schedule, launching an <15 measles and rubella campaign to increase its coverage, and introduce a combined measles-rubella (MR) vaccine to the country.

### What this study adds

- The study provides a timeline into the progress of Kenya, Africa and the world towards the control of measles through vaccination;
- The study provides detail into the innovations in measles control done in Kenya, to give an overview of what has been done, what is being done right now, and what the future holds for the fight against measles in Kenya;
- The study highlights each innovation through both the peer-review research publication on the innovation and through official government or international agency’s policy. This provides both the scientific justification of the innovations and the official actions taken by the governing bodies.

## Competing interests

The authors declare no competing interest.
